# Quantitative assessment of systolic and diastolic function in patients with systemic amyloidosis

**DOI:** 10.1186/1532-429X-18-S1-P314

**Published:** 2016-01-27

**Authors:** Daniel Kuetting, Darius Dabir, Rami Homsi, Julian A Luetkens, Hans Schild, Daniel K Thomas

**Affiliations:** Radiology, University of Bonn, Bonn, Germany

## Background

Systemic amyloidosis is a rare multisystem disease caused by extracellular accumulation of fibrillar proteins, leading to loss of normal tissue architecture and function. In clinical routine myocardial affection is assessed by late gadolinium enhancement. The aim of this study was to investigate whether patients with systemic amyloidosis (AL), both with and without cardiac affection and a healthy control group could be differentiated based on FT based systolic and diastolic strain parameters

## Methods

17 Patients with systemic AL (mean age 63. ± 11.53 years; mean LVEF 64.4 ± 9 %), 17 Patients with systemic AL and cardiac magnetic resonance (CMR) late gadolinium confirmed cardiac affection (mean age 66.5 ± 12.6 years; mean EF 52.6 ± 11 %) and 10 healthy subjects (mean age 60 ± 8.9; mean EF 60.4 ± 9.2%) %), were scanned in supine position on a clinical 1.5 T MRI scanner (Philips Ingenia. Short axis slices as well as horizontal long axis views were acquired using standard SSFP-sequences. Standard CMR parameters as well as FT derived systolic and diastolic circumferential and longitudinal strain parameters were measured.

## Results

In AL patients with cardiac affection peak systolic longitudinal strain (-13.4 ± 1.9% vs 19.9 ± 1.8% ; p < 0.05), as well as the early diastolic strain rate (EDSR) (63.69 ± 18.3% vs 75.2 ± 11.9 %, p < 0,05) were significantly reduced compared to AL patients lacking cardiac affection. Peak systolic longitudinal strain in AL patients with cardiac affection was significantly reduced in comparison to the healthy control group (20.3116 ± 1.8 p < 0.0001). Both AL patients with and without cardiac affection show significantly reduced EDSR in comparison to the healthy control group (87.14 ± 16.6; p = 0.0028 and p = 0.03). A positive correlation between IVSD and longitudinal strain (r = 0.71) was found.

Peak diastolic strain rate derived from longitudinal strain (AL cardiac: 1.17 ± 0.4 vs. AL non cardiac: 1.2 ± 0.4 vs. control: 1.3716 ± 0.4), peak systolic strain rate derived from longitudinal strain (AL cardiac: -1.06 ± 0.42 vs. AL non cardiac: -1.13 ± 0.26 vs. control: -1,3366 ± 0.1) mean circumferential strain (AL cardiac: -20.4 ± 5.8 % vs. AL non cardiac: -23.8 ± 4.5% vs. control -23.2086 ± 2.3378) and peak diastolic strain derived from circumferential strain( AL cardiac: -1.24 ± 0.3 vs. AL non cardiac: 1.4 ± 0.4 vs. control: 1.3 ± 0.8) did not differ significantly between the investigated groups.

## Conclusions

Both AL patients with and without cardiac affection showed decreased longitudinal strain and EDSR in comparison to a healthy control group, while systolic function was preserved.

